# Pilot Plant Test of Single-Pass Electrodialysis Reversal System

**DOI:** 10.3390/membranes16040114

**Published:** 2026-03-25

**Authors:** Marian Turek, Ewa Bernacka, Krzysztof Mitko

**Affiliations:** 1 Department of Inorganic, Analytical Chemistry and Electrochemistry, Faculty of Chemistry, Silesian University of Technology, ul. B. Krzywoustego 6, 44-100 Gliwice, Poland; krzysztof.mitko@polsl.pl; 2 PolymemTech Sp. z o.o., ul. Królewska 18, 00-103 Warszawa, Poland; ebernacka@polymemtech.pl

**Keywords:** electrodialysis reversal, single-pass mode, pilot plant test

## Abstract

Increasing the recovery in electrodialysis desalination may be achieved using a single-pass operation at different linear flow velocity values in the diluate and concentrate compartments. The risk of inner leakage as well as membrane bulging and damage can be minimized by controlling the pressure difference between the diluate and concentrate compartments. This solution has been tested in a pilot plant for initial demineralization of river water using an electrodialyzer of our own design. Both under- and overlimiting regimes have been tested, as well as long work cycles between electrode polarity reversals. Water with a conductivity of about 500 µS/cm was desalinated at a recovery of 70–75%, and the desalination degree was 75–96%. It was also found that the unit cost could be decreased by 52% compared to a commercial solution when the diluate conductivity was 74.3 μS/cm. A deep demineralization, from 511 μS/cm down to 17.9 μS/cm in a single-stage EDR or 8.52 μS/cm in a two-stage EDR, was also confirmed experimentally at the pilot scale.

## 1. Introduction

Electrodialysis reversal (EDR) is a modification of electrodialysis (ED) in which periodic reversal of the electrodes’ polarity allows for continuous, self-cleaning operation at high recovery rates. The electric field is reversed to remove sparingly soluble salts deposits from the membrane surface before the materials became permanently attached. Reversing the DC field eliminates the need to dose acid or antiscalants for the desalting process. Not having to apply chemicals is a major advantage of EDR over reverse osmosis (RO). EDR is used to treat brackish waters with moderate total dissolved solids (TDS) and waters that have high scaling potential [1] due to elevated levels of individual contaminants such as barium (Ba) and strontium (Sr). EDR is also effective for feed waters with a high silica (SiO_2_) content [2]. EDR can be applied to mitigate the loss of process efficiency observed when calcium, barium, and iron are present in the feed water [3]. This method is also useful for desalination of waters with a relatively high organic and colloidal content [4]. Electrodialysis reversal was successfully applied in groundwater treatment [5,6]. Thanks to its resistance to scaling and fouling, EDR can be used to treat difficult liquids like RO brine [7] or petrochemical wastewater [8]. EDR operating in single-pass mode shows particularly high resistance to scaling [9]. EDR requires less maintenance and cleaning downtime than RO [10]. EDR is able to operate with a Silt Density Index (SDI_5_) average of 12 compared to 3 for RO systems [11]. EDR systems are capable of operating with a continuous free chlorine residual of up to 1 ppm, whereas RO systems require a dechlorination process to protect the membrane [11]. EDR’s rugged thick membrane technology can ensure a membrane life of 7 to 10 years, whereas RO’s membrane is designed for 5–7 years due to the membrane’s sensitivity to various operating factors [11]. In the case of brackish water, EDR can be cheaper than reverse osmosis [12]. When comparing RO and EDR, one has to keep in mind that the basis of the process is different: in reverse osmosis, the energy is proportional to the amount of solvent that needs to be transported across the membrane; in electrodialysis, the required energy is proportional to the amount of ions that need to be transferred. Because brackish water desalination requires the removal of a relatively small amount of salt, EDR can be economically competitive [13]. The application of electrodialysis reversal in brackish water desalination has been widely studied, including treatment of river water [14] or wastewater treatment plant effluent [15]. A recent review of electromembrane treatment technologies showed that ED costs range from $0.25 to $1.20/m^3^, although the feed water composition strongly affects the overall costs [16]. All those factors justify application of EDR instead of RO for demineralization of water in the energy industry.

In electrodialysis, the product is usually desalinated water, or diluate. As a rule, it is important that the diluate recovery is high, i.e., the volume of produced diluate is much larger than the volume of concentrate. In the case of industrial applications, most often the electrodialysis process is carried out continuously and with a similar or equal thickness of the diluate and concentrate compartments. In such a case, a high diluate yield requires electrodialyzer operation either in a feed-and-bleed mode [17] or at different values of the linear flow velocity in the diluate and concentrate compartments. A higher flow velocity in the diluate compartment is required if the diluate is the product. Since the hydraulic pressure drop is proportional to the linear flow velocity, the difference between the diluate/concentration flow velocity causes a significant pressure difference between the adjacent compartments (diluate and concentrate). The pressure difference can cause bulging of the membranes and their damage. In industrial electrodialysis plants, especially electrodialysis reversal (EDR) operating at high diluate yields, recirculation of a part of the concentrate (feed-and-bleed mode [17]) is used to ensure similar linear flow velocity values in the diluate and concentrate compartments.

For example, at the Stalowa Wola EDR plant [18], river water, after coagulation and filtration, feeds the diluate compartment in the single-pass mode and the concentrate in the feed-and-bleed mode. The basic EDR line in the plant has a maximum capacity of 30 m^3^/h and consists of three electrodialyzers working in series (three-stage treatment process). Each electrodialyzer is equipped with 600 membrane pairs. There are four parallel EDR lines, resulting in a maximum capacity of 120 m^3^/h. The concentrate discharge from the system is about 15–36 m^3^/h. The pressure drop across three membrane stacks is 160–180 kPa, and the pressure difference between the diluate and concentrate compartments is 5.7–25 kPa. The inconvenience of the above solution leads to the necessity of consuming additional energy for pumping the recycled concentrate, which increases the unit energy consumption. Moreover, the residence time of the species in the concentrate is many times longer than in the case of single-pass operation, which increases the risk of scaling, i.e., crystallization of sparingly soluble chemical compounds that may foul and block the ion-exchange membranes. This is caused by the slow crystallization kinetics of the species responsible for the scaling. For example, gypsum solutions at 140% saturation can be stable for days, but at 400% saturation, gypsum precipitates in minutes. In the feed-and-bleed configuration, part of the supersaturated concentrate is recycled, and the growing crystal nuclei can spend a long enough time in the electrodialyzer to form a scale layer on the membrane.

In this paper, we describe pilot-scale research on EDR used for the demineralization of river water (pretreated with coagulation and ultrafiltration) for the purposes of a power plant. The EDR used in the research was a novel unit constructed specifically for the task. The novelty lied in the application of spacers with an uncommon design (neither a box- nor diamond-type mesh but a hexagonal one [19]—to the best of our knowledge, this type of spacer mesh is not used at all in the industry) and a novel operating procedure [20,21], which allows for working at different linear flow velocity values in the diluate and concentrate compartments without the risk of inner leakage and without the risk of membrane bulging and damage. The proposed method consists of minimizing the pressure difference between the diluate and concentrate compartments by throttling the outflow of the two media, whose flow rate is lower. We are not aware of any commercial of research system that has attempted to control transmembrane pressure in this way. The industrial practice is to keep the flow rate of diluate and concentrate similar and to apply feed-and-bleed mode on the concentrate side if high recovery is required. Our solution allows EDR to be operated in true single-pass mode with respect to both diluate and concentrate.

The novel unit offers a clear advantage over commercial EDR solutions, which typically operate in a feed-and-bleed mode: as the concentrate is not recirculated to maintain similar flow rates on both sides of the membranes, it is easier to keep the hydraulic residence time short enough to wash out the growing crystal nuclei of sparingly soluble salts before they precipitate in the concentrate compartment. Thus, the risk of scaling is lower compared to the commercial unit.

The goal of the research was to answer the following questions:

(1) Can the novel EDR unit reach a demineralization level comparable or lower than commercial EDR units used in the energy industry?
(2) Can the novel EDR unit offer a water recovery that is comparable or higher than commercial EDR units used in the energy industry?
(3) Can the novel EDR unit achieve both those feats while still being cheaper than commercial EDR units?

The research has been divided into four parts:

(1) Description of the novel elements of the EDR unit,
(2) Design process of the EDR unit with auxiliary bench-scale tests on smaller units,
(3) Pilot tests of the EDR unit to determine the stability of the operation and to observe the achievable demineralization level, recovery, and occurrence of scaling,
(4) Economic assessment of the EDR unit based on data collected during the pilot-scale test.

## 2. Materials and Methods

### 2.1. Description of Novel EDR Unit

The test EDR unit relies upon two innovations: improvement in the pressure control strategy, described in patent [20], owned by Silesian University of Technology, and [21], co-owned by Silesian University of Technology and Tauron S.A. (owner and the Łagisza and Stalowa Wola power plants); and a new design of intermembrane spacers, described in patent [19], co-owned by Silesian University of Technology and Polymemtech Sp. z o.o. The following chapter describes those two elements in detail. The electrode and membrane materials were not the subject of innovation in the presented research.

#### 2.1.1. Pressure Control Strategy

**Figure 1:**
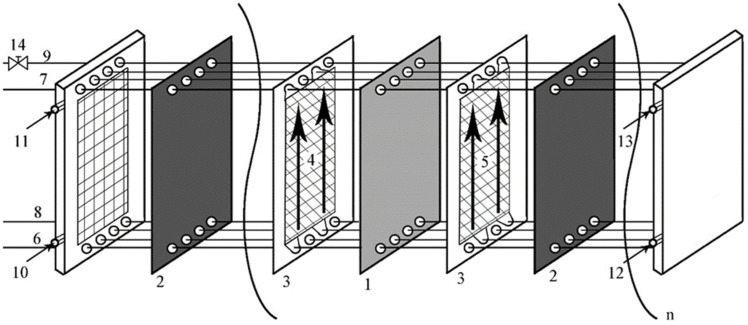
General scheme of an electrodialysis stack [20,21]: anion-exchange membranes (1), cation-exchange membranes (2), spacers (3), diluate (4), concentrate (5) compartments, diluate inlet (6), diluate outlet (7), concentrate inlet (8), concentrate outlet (9), catholyte inlet (10), catholyte outlet (11), anolyte inlet (12), anolyte outlet (13), throttling valve (14). Arrows depict the direction of the liquid flow.

**Figure 2:**
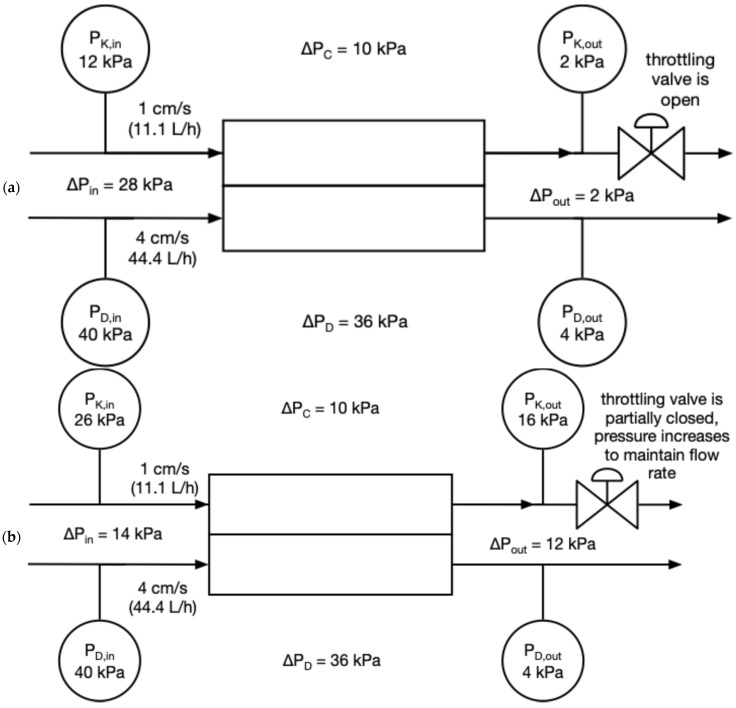
Scheme of pressure regulation strategy: (**a**) operation without throttling, where the pressure difference between diluate and concentrate at the inlet is over 25 kPa, creating a risk of membrane bulging; (**b**) concentrate outlet is throttled, where the concentrate inlet pump must work at a higher pressure to maintain the flow rate, which causes the pressure difference between diluate and concentrate to not exceed 25 kPa.

The general scheme of an electrodialysis stack is presented in Figure 1. A plate-and-frame electrodialysis module is equipped with alternately arranged anion- and cation-exchange membranes, separated by spacers that determine the inter-membrane distance and thus the thickness of the diluate and concentrate compartments. The diluate and concentrate compartments of the electrodialyzer are fed co-currently. To minimize the pressure difference between the diluate and concentrate compartments and to avoid the risk of membrane bulging, the concentrate outflow is throttled with a valve. An example of the flow throttling strategy is presented in Figure 2. The volumetric flow rate is kept constant. When the concentrate outflow is unthrottled (Figure 2a), the difference in the flow rates in the concentrate and diluate compartments creates the difference in pressure—in the presented example, the registered pressure is 40 kPa at the diluate compartment inlet and 12 kPa at the concentrate compartment inlet. This creates a pressure difference of 28 kPa, which exceeds the safety limit of 25 kPa, meaning there is a risk of membrane bulging. The value of the safety limit, 25 kPa, was chosen because it is the maximum allowable pressure difference for industrial-scale electrodialysis used in the Stalowa Wola plant [18]. To prevent the negative effects of pressure difference, the concentrate outlet is throttled (Figure 2b), meaning that while the volumetric flow rate is kept constant, the inlet pump must work at high pressure, increasing the pressure throughout the entire concentrate compartment. The throttling increases the concentrate pressure, which means that the difference between diluate and concentrate pressure is lowered from 28 kPa down to 14 kPa, well below the safety limit. In practice, the adjustment strategy is to (1) keep the pressure of the concentrate inlet at a value of 0.3–0.7 of the diluate inlet pressure and (2) not exceed a value of 150 kPa of the concentrate outlet pressure. Throttling the outlow is performed by a valve. Constriction of the flow cross-section means that the pressure must be increased to maintain the volumetric flow rate, which in effect changes the pressure differences between the different points of the electrodialyzer.

#### 2.1.2. Novel Intermembrane Spacers

**Figure 3:**
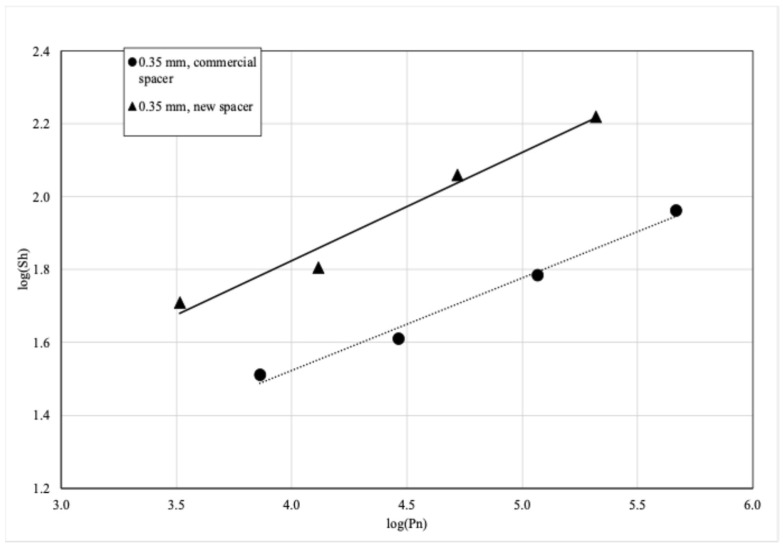
Comparison of a new spacer to the commercial spacer of similar thickness, adapted from [19]. The specific relationships between Sh and Pn for different spacers were determined in previous research [19] and were not tested separately in the pilot plant discussed here.

By using thin spacers, not previously used in industry, electrodialysis can be carried out in a single-pass mode of the solutions through the module—in contrast to a typical EDR, where the concentrate is recirculated. The positive effect of a new spacer was demonstrated in preliminary research [19] and can be seen in Figure 3, which shows the relationship between the Sherwood number (Sh, a dimensionless number describing the mass transfer) and power number (Pn, a dimensionless number describing the power consumption/pressure drop), given as [22]:

(1) Sh = k · h/D,

(2) Pn = (ΔP · u/L) · ρ^2^ · h^2^/μ^3^,

where k denotes the mass transfer coefficient, h is the spacer thickness, D is the diffusion coefficient of the species in the water, ΔP is the pressure drop, u is the linear flow rate, L is the flow channel length, and ρ and μ are the fluid density and dynamic viscosity. In the case of electromembrane modules, the mass transfer coefficient (and by extent, the Sherwood number) can be directly linked to the limiting current density, i_lim_ [23]:

(3) Sh · D/h = k = i_lim_(t_m_ − t_s_)/(zFC_s_^d^),

where t_m_ and t_s_ denote the ion transport number in the membrane and water phases, respectively, z is the valence number of the ion, F is Faraday’s constant, and C_s_^d^ is the diluate concentration.

**Figure 4:**
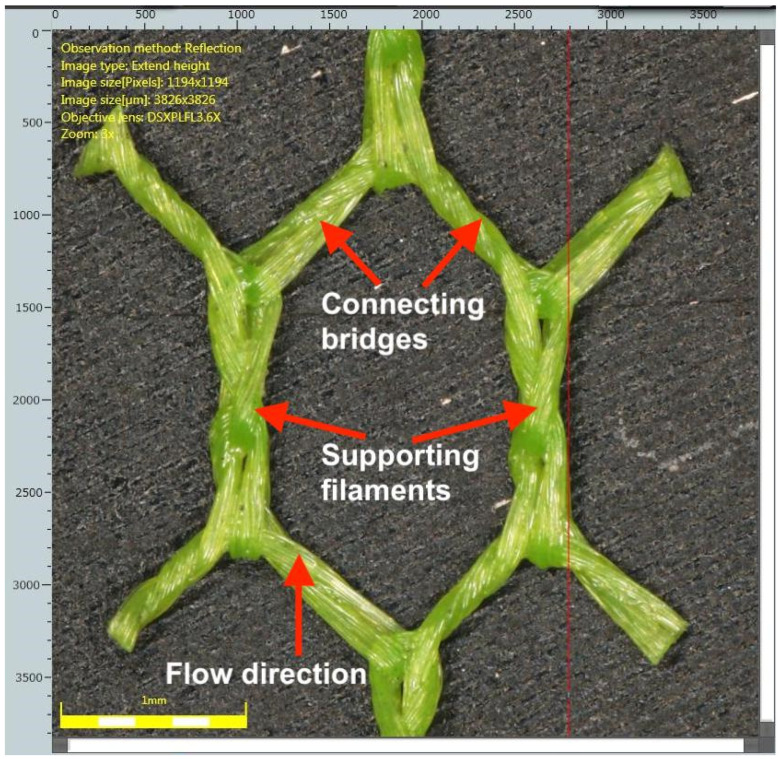
Custom-made hexagonal 0.35 mm spacer used in the electrodialysis stack.

The log–log plot of Sh vs. Pn clearly shows a separate line for each spacer type. In this frame of reference, the upper-left corner depicts the best performance of the spacer—high mass transfer coefficient (high Sh) at low hydraulic pressure drop (low Pn)—whereas the bottom-right corner depicts the worst-performing spacer—high pressure drop and low mass transfer coefficient. The novelty of the new spacer lies in its mesh design: whereas the industry typically uses either box- or diamond-type meshes, the spacer used here has a three-dimensional shape made of polygons with a number of sides being higher or equal than 5 (specifically, a hexagonal mesh made by knitting polyamide fibers was used in this research—see Figure 4). The increase in mass transfer is caused by the connecting bridges between the support filaments. The connecting bridges, themselves having the shape of a cylinder twisted along its longitudinal axis by 90°, obstruct the flow in a way that aids mixing between the boundary diffusion layer and the bulk of the flowing solution. This additional mixing increases the mass transfer coefficient, increasing the value of Sh compared to traditional spacers operating at the same flow rate (Pn). The specific geometric considerations are discussed in the US patent [19] that is co-owned by the Silesian University of Technology and Polymemtech Sp. z o.o., institutions employing the authors of this work.

Additionally, the novel spacer exhibits low residence time variance (e.g., low variance of the E(t) residence time distribution obtained by the stimulus-response method of RTD determination). A low variance indicates the lack of dead zones, which makes it possible to operate at high supersaturation of the concentrate with respect to sparingly soluble salts, which significantly increases the possible water yield. Thus, the ED can produce a smaller volume of a more saline concentrate, compared to the case when the concentrate salinity is limited by the concentration of sparingly soluble salts. In the presented work, however, we did not test the specific relationships of dimensionless numbers, nor did we optimize the spacer geometry further, as it was deemed out of the scope of the pilot-scale tests of demineralization.

### 2.2. Auxiliary ED Experiments During the EDR Design Step

As a first step, the required membrane length for the single-pass electrodialyzer was investigated using the methodology presented in [24] by assuming that the electrodialyzer should desalinate river water with a conductivity of 500 µS/cm down to 35 µS/cm. The required membrane length, L, was calculated using the following equation [24]:

(4) L = [(C_L_ − C_0_)/(i_L_ − i_0_)] · suzF · ln(i_0_/i_L_),

where C_L_ are C_0_ are the molar concentrations of Na_2_SO_4_ solution corresponding to 500 and 35 µS/cm, respectively, i_L_ and i_0_ are 80% of the experimentally determined limiting current density for concentration *C_L_* and *C_0_*, respectively, the linear flow velocity is *u*, z is the valence of the ion being transported across the membrane, F is Faraday’s constant, and *s* is the spacer thickness. The equation is based on the fact that the current density distribution along the membrane is not linear but instead resembles a first-order kinetic equation, with the position along the electrodialyzer being equivalent to time [24,25,26,27,28,29]. The determination of the limiting current density was performed in a batch-mode electrodialyzer equipped with 4 membrane pairs (Ionsep AM-A/AM-C membranes, effective membrane area: 4.2 cm^2^) using model solutions.

Next, the possibility of reaching the required diluate conductivity was experimentally verified in a bench-scale batch-mode electrodialyzer with a 4.2 cm^2^ effective membrane area equipped with four pairs of Ionsep AM-A and AM-C membranes and our own 0.35 mm thin spacers. The experimental verification was conducted using real river water. For the batch studies, the time it took to reach a set conductivity was equivalent to the required membrane length in continuous-mode operation. Given infinite time, it should theoretically be always possible to reach desired salinity, just as an infinitely long membrane working in a single-pass mode could always reach a desired salinity (assuming no diluate limit due to low conductivity of highly diluted solutions or no concentrate limit due to water transport). In [24], the methodology for calculating the required membrane length, used to derive Equation (4), was based on the assumption that the current density distribution along the electrodialyzer is not linear; instead, the current density *i*(*l*) at position *l* along the membrane follows a first-order kinetic equation. The equivalent time required for batch processing can be derived using the same approach as in [24] except by assuming that the current density is a function of the process time and not the position along the membrane:

(5) i(t) = i_0_ · exp(−k · t),

where k can be written as a function of the current density at the beginning of the process (i_0_), at the end of the process (i_L_), and the time T required to reach the required salinity:

(6) k = 1/T · ln(i_0_/i_L_),

Knowing the volume of the diluate solution, V, the initial and final concentrations, C_0_ and C_T_, the number of membrane pairs, x, and the effective membrane area, A, the required electric charge can then be calculated assuming 100% current efficiency, 1:1 salt, and insignificant volume change:

(7) q = (C_0_ − C_T_)VF/x = ∫_0_^T^ [i_0_ · exp(−1/T · ln(i_0_/i_L_) · A · t] dt,

where F is Faraday’s constant. After solving the integral and rearrangement, Equation (4) becomes the formula for the required batch processing times:

(8) T = [(C_0_ − C_T_) · V · F · ln(i_0_/i_L_)]/[x · i_0_ · A · (1 − i_L_/i_0_)],

### 2.3. Pilot-Scale Test of the Constructed Novel EDR Unit

**Figure 5:**
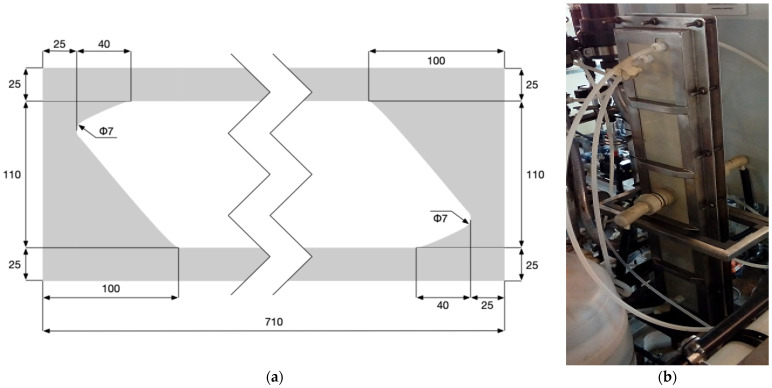
Electrodialyzer used in the pilot-scale tests: (**a**) spacer dimensions [mm]; (**b**) assembled stack.

**Figure 6:**
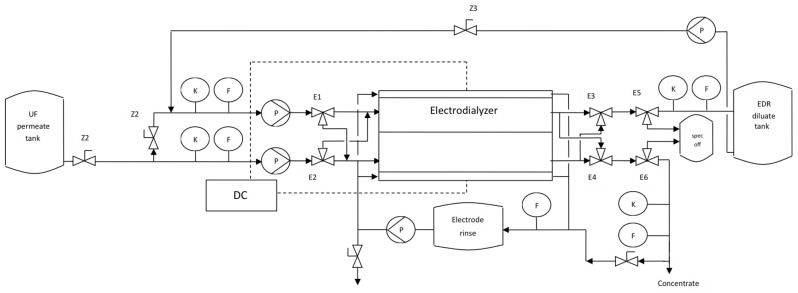
Process flow diagram of the electrodialysis reversal pilot; DC—YK-AD5050 power supply (0–50 VDC, 0–50 A, 2500 W), K—Condumax CLS21D/CLS15D-B1A1 conductivity sensors, F—analog flow meters, Z—manual valves, P—March TE-6T-MD pump, E—three-way electrovalve (type 0330-T-04).

Next, an electrodialyzer of our own design was built. The novel EDR unit was equipped with eight pairs of Ionsep AM-A and AM-C membranes and our own 0.35 mm thin spacers [19]. The spacer mesh was fabricated using a knitted technology and was integrated with the gasket by thermo-screen printing. The electrodialyzer and the spacer dimensions are shown in Figure 5, while the process flow diagram of the plant is presented in Figure 6. The electrodialyzer has dimensions of 91 cm x 16 cm and an effective membrane length of 76 cm, which—with a small inter-membrane distance (0.35 mm)—ensured that the desired degree of diluate desalting, as well as the desired concentrate concentration, could be achieved in the “single-pass” mode. The width of the compartments was 11 cm, and the effective membrane area was 0.0836 m^2^.

### 2.4. Economic Analysis

Finally, an economic analysis of the proposed solution was performed with the following assumptions: membrane price *C_m_* of 38 €/m^2^, EDR plant equipment cost *C_eq_* of 321€ per 1 m^2^ of installed membrane [30], membrane life time *t_life_* of 80,000 h, effective membrane area *η_m_* of 75%, AC/DC rectifier efficiency *η_AC/DC_* of 90%, pump efficiency *η_p_* of 80%, and electric energy cost *C_el_* of 0.11 €/kWh. For comparison, the energy costs and investment costs were calculated the same way as for the EDR plant in Stalowa Wola, Poland, which desalinates water from the San river [18]. The following equations were used:

(9) E_DC_ = (n · U · A · i)/(η_AC/DC_ · V_F_),

(10) E_AC_ = (V_D_ · ΔP_D_ + V_C_ · ΔP_C_)/(η_p_ · V_F_),

(11) CAPEX = [2n · (C_m_ + C_eq_) · A/η_m_]/(t_life_ · V_F_),

(12) OPEX = C_el_ · (E_AC_ + E_DC_),

where *n* is the number of membrane pairs, *U* is the voltage drop per membrane pair, *i* is the current density, *V_F_* is the flow rate of feed water entering the electrodialyzer, *V_D_* and *V_C_* are the flow rates of the diluate and concentrate, respectively, in the electrodialyzer (in the case of Stalowa Wola’s feed-and-bleed configuration that includes recycling the concentrate), *A* is the membrane area, *E_DC_* is the DC electric energy required for the desalination, and *E_AC_* is the electric energy required for pumping the solutions. Appendix A contains a step-by-step description of the procedure for the calculation of OPEX and CAPEX.

## 3. Results

### 3.1. Design of Single-Pass Electrodialyzer and the Auxiliary ED Experiments

In order to design the electrodialysis process according to the proposed methodology, one must determine the limiting current density for two cases, simulating two ends of a long single-pass electrodialysis process. The methodology follows our previous work [24] and can be summarized as follows:

- Assume the design of a counter-current, single-pass electrodialyzer that works on a solution with a conductivity of 800 µS/cm. The feed is desalinated down to 35 µS/cm in the diluate compartment; simultaneously, the concentrate conductivity increases from 800 to 3000 µS/cm,
- Consider two ends of said counter-current electrodialyzer. At one end, the 800 µS/cm solution enters the diluate compartment, while the 3000 µS/cm concentrate leaves the concentrate compartment. At the other end, the 35 µS/cm solution leaves the diluate compartment, while the 800 µS/cm feed enters the concentrate compartment,
- Determine the limiting current density in a bench-scale electrodialyzer operating in a batch mode on a 35 µS/cm solution in the diluate compartment and 800 µS/cm in the concentrate compartment. This will simulate one end of the counter-current electrodialyzer,
- Determine the limiting current density in a bench-scale electrodialyzer operating in a batch mode on an 800 µS/cm solution in the diluate compartment and a 3000 µS/cm solution in the concentrate compartment. This will simulate the second end of the counter-current electrodialyzer,
- Assume working at 80% of the limiting current density and a first-order kinetic model for the distribution of the current density along the membrane; use Equation (1) to calculate the required membrane length.

Table 1 presents the determined values of 80% of the limiting current density for both cases as well as the estimated membrane length required to a reach diluate conductivity of 35 µS/cm. The required batch processing time was calculated assuming four membrane pairs, a 4.5 cm^2^ effective membrane area and a 55 cm^3^ diluate volume (the parameters of the bench-scale verification experiment are discussed in the next section). The limiting current density was determined in a batch ED unit using the same spacer/membrane combination that was later used in the pilot EDR unit using the abovementioned methodology of simulating a long single-pass ED process. Based on the limiting current density obtained in these tests, Equations (4) and (8) allowed for calculating the required length L and time T. The results suggest that it should be possible to reach the required degree of desalination in the reasonably short electrodialyzer (L = 0.68 m at a linear flow velocity of 4 cm/s) or within a reasonably short time (T = 47 min at a linear flow velocity of 4 cm/s).

**Figure 7:**
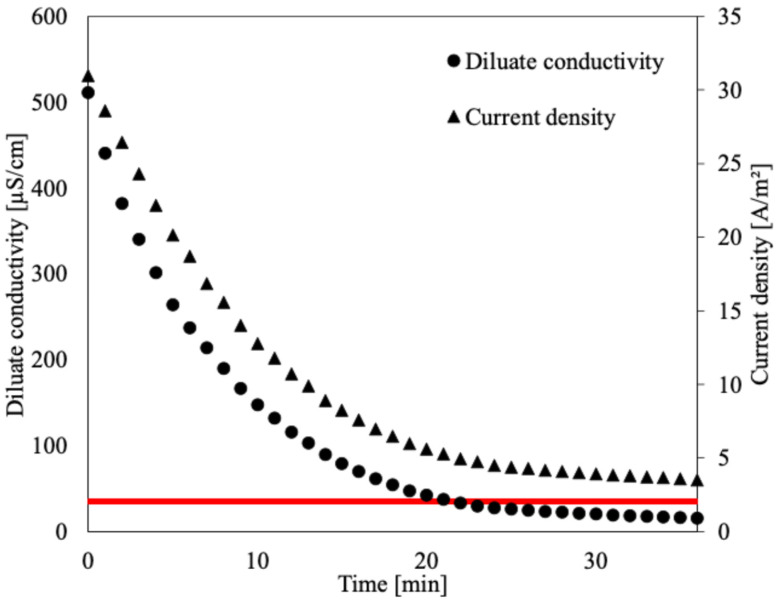
Results of bench-scale verification (singular ED batch of river water) of deep demineralization by electrodialysis. Red line denotes the target 35 µS/cm conductivity.

The next step was to confirm whether the electrodialysis process can reach the low diluate conductivity envisioned for the single-pass operation. Generally, the process of electrodialysis is subjected to two boundaries that limit the concentration of the product. The upper boundary stems from the transport of water accompanying ion migration across the ion-exchange membranes. For example, if 19 moles of water are transported per 1 mole of salt, the salinity of the produced concentrate will never exceed a molar fraction of 1/20 regardless of other process conditions. The lower boundary, much more important for the investigated river water desalination case, stems from the fact that as the concentration of the produced diluate decreases, the electric resistance of the membrane stack increases, meaning a higher voltage drop, and more energy is thus required to maintain the process. Eventually, the required voltage drop may become impractically high, necessitating modifications to membrane stack (e.g., electrodeionization) to produce cleaner water. In this study, using the bench-scale preliminary experiments made before construction of the pilot-scale ED, a constant voltage of 7 V per membrane stack (1.75V per membrane pair) was maintained. Figure 7 presents the change in conductivity and current density during the bench-scale test of a single ED batch run. Thanks to the higher applied voltage drop per membrane pair, the low intermembrane distance and the good mass transfer properties of the applied spacer, it was possible to not just reach the target 35 µS/cm diluate conductivity but go deeper, down to 15.4 µS/cm. The results show that it is possible to reach a very low conductivity of water solely by electrodialysis and to decrease the load on downstream methods (e.g., electrodeionization/ion exchange) if further polishing is required.

The initial estimation of the required batch processing time showed that it would require 47 min to reach 35 µS/cm at 4 cm/s (see Table 1). The results of the batch experiments show, however, that it only took 21–22 min. The discrepancy stems from the higher current density than the one determined during the design stage (the batch-mode experiment was performed using real river water, not an 800 µS/cm model solution). If the real experimental values of the current density obtained at 35 µS/cm and at the beginning of the experiment (4.95 and 31 A/m^2^, respectively) are put into Equation (8) (31 and 3.5), the required batch processing time T is 23 min, which matches very closely the experimental data.

### 3.2. Pilot-Scale Tests of Single-Pass EDR

Finally, pilot-scale tests were conducted at the Łagisza power plant in Będzin, Poland, on feed water (river water subjected to coagulation/ultrafiltration) with the parameters presented in Table 2. The feed water in question came from the Przemsza river and was subjected to coagulation/ultrafiltration before it was fed to the EDR unit. The daily results of the electrodialysis tests are presented in Table 3. The EDR worked in continuous mode for the entire day, and the electrode polarity was periodically reversed throughout the day (the time between the polarity switch was equal to the sum of the working time and spec-off time). The operation was performed in constant-current mode in a single-pass setup, with flow rates in the laminar flow regime. The range of the tested volumetric flow rate was set to not exceed 4 cm/s as the linear flow velocity to keep the pressure drop sufficiently low (see Figure 2). The current was kept at the level required to reach a diluate conductivity that was the same as or lower than the conductivity of the Stalowa Wola demineralization station product.

During the experiments, the recovery was changed daily at a constant working/spec-off time ratio to test the stability of the operation under set conditions. Once it was determined that the system was stable (i.e., no scaling for the day of continuous operation), the working/spec-off time ratio was increased to check if higher recovery could be achieved under stable conditions without scaling. The recovery was calculated as:

(13) Y = (t_work_ · V_D_)/[(V_D_ + V_C_) · (t_work_ + t_spec-off_)] · 100%,

where V_D_ is the daily average flow rate of the diluate (product), V_C_ is the daily average flow rate of the concentrate (waste), t_work_ is the length of the work cycle, and t_spec-off_ is the length of the spec-off cycle.

It was confirmed that the single-pass, continuous electrodialysis could work with different linear flow velocities in the diluate and concentrate compartments. Deep demineralization was achieved (diluate conductivity < 35 µS/cm). On two occasions, a two-stage EDR was simulated by collecting the regular diluate and using it as a feed for the next day. On these occasions, a very low conductivity of the diluate was achieved, at 8.52 and 13.3 µS/cm.

### 3.3. Economic Analysis of the Proposed Solution

Table 4 presents the calculated energy consumption (both in terms of energy consumed by the DC power source for the EDR unit and the energy consumed by the pumps that feed the EDR unit) and investments costs in comparison with the Stalowa Wola EDR plant at a given flow rate and conductivity of the process streams, product recovery, and pressure drop. Two cases were chosen as the basis for economic comparison: (1) the average of all measurements with a working/spec-off time ratio similar to that of Stalowa Wola (17:1.5 case presented in Table 3), and (2) the deep demineralization case. The first case was selected to showcase the application that was most similar to the commercial EDR unit in terms of the degree of demineralization and recovery, while the second case was selected to showcase the application producing a better quality of the product. In the case of the proposed solution, the majority of energy consumption was used for operating the electrodialysis process, with a smaller fraction being used for pumping. This is a result of working at a relatively high voltage drop. The Stalowa Wola costs are in the range of typical costs reported for ED (0.25–1.2 $/m^3^ [16]), a result of the relatively low conductivity of the feed water, while the costs estimated for the novel EDR unit are below the range of the reported ED costs. The novel EDR unit can also be operated at a high voltage drop (7 V), achieving deep demineralization (17.9 µS/cm diluate conductivity). In this case, the unit costs are 30% higher than in the “average” case (i.e., when the diluate conductivity is comparable with the commercial EDR), but they are still 37% less than commercial EDR while delivering a better quality of the product.

To analyze the sensitivity of the proposed economic model, following variables were investigated:

- Membrane price: A value of 38 $/m^2^ was assumed for the base scenario, following real prices of cheap ion-exchange membranes [31], 10 $/m^2^ for the optimistic scenario, and 189 $/m^2^ for the pessimistic scenario. While the 10 $/m^2^ value is nowhere near commercial prices and we disputed this value in our previous work [32], a lot of techno-economic analyses claim that the membrane cost can be lowered down to this level. The pessimistic scenario was conducted to assume the price of high-end homogenous membranes following the 222 $/m^2^ data presented in [30] with an exchange rate of 1 $ = 0.85 €.
- Alectricity price: A value of 0.11 €/kWh was assumed as the base scenario, following real prices of electricity for non-household consumers in Poland [33]. For the optimistic scenario, an electricity price of 0.05 €/kWh was assumed following the lowest electricity price for non-household consumers in the EU in 2024 (Norway) [33], and for the pessimistic scenario, a value of 0.26 €/kWh was assumed (highest price, Ireland),
- EDR equipment: The base scenario was 321 €/m^2^, following Nayar et al.’s [30] assumption of an ED plant CAPEX of 600 $/m^2^, out of which 222 $/m^2^ is the membrane and 378 $/m^2^ is everything else, and assuming a currency exchange of 1 $ = 0.85 €. The optimistic scenario was assumed to be 20% lower than the base scenario, while the pessimistic scenario was assumed to be 20% higher.
- Membrane lifetime: The base scenario was 80,000 h of work, which is an assumption based on our experiences with industrial EDR plants. For sensitivity analysis, +20% and −20% lifetime values were assumed as the optimistic and pessimistic scenarios, respectively.

The results of the sensitivity analysis for the “average” case, when the quality of the product is comparable between novel EDR (74.3 μS/cm) and commercial EDR (85 μS/cm), are presented in Table 5. The results of the sensitivity analysis for the “deep demineralization” case, when the quality of the novel EDR product is significantly higher than the quality of a commercial EDR unit, are presented in Table 6. The analysis for the average case, when the quality of the product is comparable to commercial EDR, shows that in almost every case, the total costs estimated for the commercial EDR working in the Stalowa Wola power plant are lower than the estimated costs of the EDR tested in the Łagisza power plant. Only when all the analyzed factors are changed simultaneously does the pessimistic scenario for Łagisza EDR approach the base scenario for Stalowa Wola EDR. The results suggest that even a significant (20%) decrease in membrane lifetime would be economically justifiable, as long as the equipment, membrane, and electricity costs are comparable with the ones applicable in the Stalowa Wola case. For the deep demineralization case (17.9 μS/cm product conductivity), the costs are much more sensitive to the electricity price because of the higher voltage and current density required to achieve the low diluate conductivity. In this case, an increase in electricity price from 0.11 €/kWh to 0.26 €/kWh makes the novel EDR unit more expensive than the commercial EDR unit. On the other hand, better product quality can have economic consequences downstream, for example, if the EDR diluate was to be further demineralized using electrodeionization. Downstream treatment, however, is out of the scope of this work.

## 4. Discussion

The results confirmed the possibility of deep demineralization with EDR alone, reaching a diluate conductivity as low as17.9 µS/cm in a single-stage EDR process or 8.52 µS/cm if a second stage were to be used. This is an exceptionally low salinity for conventional electrodialysis. If the diluate were to be further demineralized by electrodeionization and/or ion exchange, deep demineralization could decrease the salt load to be removed downstream. This is especially important for ion-exchange polishing of the diluate. A lower EDR diluate conductivity would increase the number of bed volumes that can pass through the ion-exchange column before regeneration is required, decreasing the chemical consumption and the volume of post-regeneration lyes. Deep demineralization was possible because a high voltage was applied, indirectly indicating that the electrodialysis process was working in the overlimiting current regime. While we do not have direct evidence of this, indirect proof comes from the high voltage drop per membrane pair. Typically, when the conductivity is low, the limiting current density is exceeded when the applied voltage is higher than ca. 2 V/pair [34]. During the EDR pilot tests, deep demineralization was reached at ca. 5–7 V/pair. If the EDR process was conducted above the limiting current density, in the so-called overlimiting current regime, it would potentially provide a smaller required footprint of electromembrane units [35]. Electroconvection in the overlimitting regime can mix the ion-depleted layer close to the membrane with the ion-rich solution from the bulk, resulting in increased mass transfer [36]. The origin of the overlimiting current varies. In the case of highly selective cation-exchange membranes, electroconvection plays a major role, while water splitting is negligible. However, in some cases, often at anion-exchange membranes, dissociated protons and hydroxide ions contribute to the overlimiting current [36]. The existence of electroconvective vortices in an electrodialyzer working in the overlimiting regime has been proven experimentally [37], and a detailed review of the role that electroconvection plays in electrodialysis can be found in [38]. The spacer geometry also plays a role in generating electroconvective flow. Spacers exhibiting dead zones at the membrane surface can lead to controlled formation of electroconvective vertices [36]. On the other hand, some tests using heterogeneous ion-exchange membranes have shown that the overlimiting current arises from a decrease in membrane permselectivity and the resulting leakage of co-ions [35] and that it does not contribute positively to desalination.

According to the results presented in [36], the inter-membrane spacer itself can promote the occurrence of electroconvective vortices, chaotic flow vortices that mix the bulk fluid with an ion-depleted fluid in the diffusion layer near the membrane surface. The intermembrane spacer used in the presented research is different to the three spacers tested in [36]—see Figure 4—so there is no obvious 1:1 comparison. Our unconfirmed working hypothesis is that connecting bridges constrict the flow in a way that at least partially shields the boundary layer (meaning the electroconvection vortices are protected from the shear flow [39]) and promotes the occurrence of electroconvective vortices. We speculate that in the pilot-scale experiments, we run the electrodialysis process in an overlimiting regime, and we are basing our speculation on the fact that the voltage drop per membrane pair was up to 7 V, higher than the 1.75 V value used in the bench-scale experiments and higher than is generally observed in underlimitting conditions [34]. The higher voltage drop allowed for reaching down to 19 µS/cm in a single-stage, single-pass module, but we did not observe anything suggesting a decreased permselectivity of the heterogenous ion-exchange membranes we used. Losing the membrane’s permselectivity should affect the separation and should make achieving a low diluate conductivity impossible, which did not happen. However, the used membrane stack did not allow for direct observation of the diluate channel in operation, so this hypothesis remains unproven, and at this point we may only speculate. Performing flow field simulations to investigate the effect of the spacer geometry may be a promising direction for future research.

**Figure 8:**
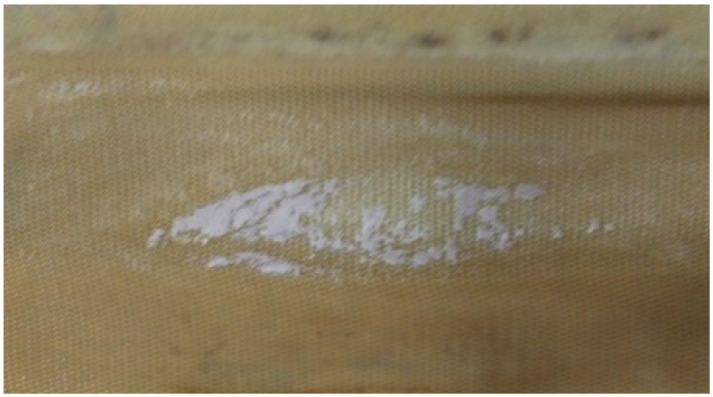
Traces of scaling on the membrane after the working time was prolonged to 81 min before electrode polarity change.

It is also worth noticing that a relatively high recovery can be achieved (69.4%, comparable with a commercial EDR unit working on a similar type of feed). The spec-off time—the time required to flush out the hold-up volume after electrode polarization change—was relatively short compared to the working time. The electrodialyzer work was stable when 1–1.5 min of spec-off time followed 5–62 min of work at a constant electrode polarity. When the working time was prolonged to 81 min, a rise in voltage was observed during the cycle (on average from 2.125 to 2.475 V per membrane pair), indicating onset scaling happening on the membrane surface. The occurrence of scaling was confirmed visually after dismantling the membrane stack—see Figure 8.

Thanks to the novel spacers improving the mass transfer, the proposed solution can achieve similar desalination to the commercial unit in Stalowa Wola at a shorter effective membrane length. The novel spacers, as discussed in Section 2.1.2, offer higher mass transfer coefficients than commercial spacers at the same hydrodynamic conditions. This means that the conversion (e.g., the demineralization degree per membrane length) is higher, which results in less membrane being needed, so the overall costs of the electrodialysis process are lower. It is worth noting that in electromembrane processes, there is always a trade-off between investment costs and operating costs. Lowering the current density can substantially decrease the energy consumption, but at a low current density, the required membrane area is high, raising not only the investment costs but also the environmental impacts related to the membrane’s production and disposal. On the other hand, working at a high current density helps to bring the membrane costs down but increases energy consumption. In the case of low-salinity water, such as river water, the investment costs play a bigger role than energy consumption, as evidenced by the results of the economic simulation.

The membrane lifetime is a separate question. The economic analysis presented here is based on the central assumption that two membranes working at the same pressure difference between diluate and concentrate should exhibit the same lifetime. It is safe to assume an 80,000 h lifetime for the membranes used in the commercial EDR station in Stalowa Wola, a plant that works in a feed-and-bleed configuration with the same flow rate in the diluate. The pressure difference in that commercial EDR station is kept below 25 kPa. Based on the central assumption that a similar pressure drop will result in similar wear and tear of the membrane, we assert that the lifetime of the membranes in the presented single-pass EDR would also be 80,000 h. Of course, the experiments presented here were only done for a few weeks, and the fact that a decreased lifetime of the membrane was not observed is not direct proof that the membrane would behave the same for the full 80,000 h. However, the sensitivity analysis of the economic model suggests that the novel EDR unit can be economically competitive with the commercial EDR solution, even if it turns out that the membrane lifetime is decreased by 20%.

## 5. Conclusions

Based on the presented results, we can confirm that the novel EDR unit can reach a demineralization level comparable to or lower than the commercial EDR units used in the energy industry, specifically the commercial EDR unit in Stalowa Wola. The novel EDR unit can offer comparable water recovery at lower costs thanks to the novel spacers having a higher mass transfer than the commercial spacers. The new pressure control method allows for reaching a product recovery in the single-pass mode that is comparable to that which the commercial unit reaches in the feed-and-bleed mode. This means that no concentrate recycle is needed, which not only decreases the pumping costs but also means, most importantly, that there is no possibility of foulants being recycled back to the EDR unit and accumulating in the concentrate compartment. While we cannot directly prove at this moment that the new pressure control system does not lower the membrane lifetime, the economic analysis suggests that our unit is still cheaper than the commercial solution even if we assume it causes a 20% decrease in the membrane lifetime.

The authors’ employers, Silesian University of Technology and Polymemtech Sp. z o.o., are continuing the research on ED and EDR modules equipped with novel spacers and on transmembrane pressure minimization as a part of an ongoing R&D project funded under the European Funds for a Modern Economy (FENG) scheme. The pilot-scale EDR described in this work is owned by Polymemtech Sp. z o.o. and is available for commercial tests. We believe the presented solution can find applications not only in the energy industry but also in the chemical industry, fertilizer industry, and food industry and in any use cases when high water recovery is required and the scaling potential is high.

## 6. Patents

The work reported in this manuscript has resulted in two patents:

Turek, M.; Laskowska, E.; Mitko, K.; Sąkol-Sikora, D.; Stawiarz, T.; Krzyżak, W.; Zdeb, J.; Smółka, W. Method of conducting the electrodialysis process, Polish patent PL241092 (2020).

Turek, M.; Laskowska, E.; Mitko, K. Electrodializer and method of conducting the electrodialysis process, Polish patent PL241481 (2020).

## Figures and Tables

**Table 1:** Estimation of the required membrane length. i_0_—80% of the limiting current density measured at the simulated one end of the electrodialyzer (35 µS/cm), i_L_—80% of the limiting current density measured at the simulated second end of the electrodialyzer (800 µS/cm), U_0_ and U_L_—voltage drops per membrane pair at both simulated ends of the electrodialyzer, respectively, measured excluding the anode/cathode compartment, L—required membrane length, T—required batch processing time.

Linear Flow Velocity [cm/s]	i_0_ [A/m^2^]	U_0_ [V]	i_L_ [A/m^2^]	U_L_ [V]	L [m]	T [min]
0.5	0.68	0.7	8.33	0.725	1.62	105
1	0.91	0.675	9.43	0.725	0.97	88
2	1.31	0.725	11.24	0.7	0.61	69
4	1.94	0.825	16.76	0.825	0.68	47

**Table 2:** Parameters of EDR feed (river water after coagulation/ultrafiltration), period of testing: 10 April 2018–21 September 2018.

Parameter	Average	Lowest	Highest
Conductivity [µS/cm]	502 ± 15	479	522
pH	7.75 ± 0.18	7.40	7.97
Turbidity [NTU]	0.28 ± 0.19	0.11	0.68
Total hardness [mmol/L]	2.38 ± 0.35	2.01	3.02
Ca^2+^ [mg/L]	65.6 ± 11.2	52.0	86.0
Mg^2+^ [mg/L]	17.8 ± 3.1	13.9	24.0
SiO_2_ [mg/L]	4.75 ± 0.95	3.31	6.66
SO_4_^2−^ [mg/L]	48.1 ± 4.2	44.0	59.5
Cl^−^ [mg/L]	30.8 ± 3.8	29.8	42.6
COD_KMnO4_ [mg O/L]	2.54 ± 0.49	2.13	3.50

**Table 3:** Daily averages observed during pilot-scale tests of the novel EDR unit, *D*—diluate, *C*—concentrate, *F*—feed water, *i*—current density, *U*—voltage per membrane pair, Δ*P*—pressure drop on the compartment.

Flow Rate [L/h]		Conductivity [µS/cm]			i [A/m^2^]	U [V]	ΔP [bar]		Working/Spec-Off Time [min]	Recovery [%]
D	C	F	D	C			D	C		
5:1.33 working/spec-off ratio—scaling not observed										
23.1	11.4	522	57.7	1463	6.6	3.5	0.5	0.2	5/1.33	52.9%
24.9	10.4	516	63.5	1599	6	3.25	0.73	0.3	5/1.33	55.7%
21.6	6.4	498	89.2	1800	4.8	2.12	0.7	0.28	5/1.33	60.9%
8:1–1.5 working/spec-off ratio—scaling not observed										
24	17.1	512	94.4	1026	6	2.5	0.8	0.6	8/1.5	49.2%
37.2	21	520	88.7	1480	7.2	1.88	0.36	0.1	8/1.33	54.8%
37.5	10.5	481	19.1	2176	11.4	7	0.36	0.06	8/1.33	67.0%
37.5	10.5	481	39.2	2059	10.8	6	0.36	0.06	8/1.33	67.0%
34.1	25.2	500	43.8	996	7.8	2.31	0.34	0.31	8/1.17	50.2%
37.5	27	523	31	1206	10.2	5.5	0.4	0.16	8/1.17	50.7%
37.2	22.6	500	56.7	1230	7.2	1.62	0.41	0.29	8/1	55.3%
17:1.5 working/spec-off ratio—scaling not observed										
40.8	24	508	35	1312	9.6	3.62	0.6	0.3	17/1.5	57.9%
45	20.5	511	17.9	1540	11.4	7	0.7	0.12	17/1.5	63.1%
42.6	18.4	502	111.6	984	7.8	1.38	0.8	0.12	17/1.5	64.2%
35.8	13.1	522	80	1730	7.2	1.12	0.7	0.16	17/1.5	67.3%
25.8	8.4	511	92.6	1796	5.4	2.12	0.8	0.3	17/1.5	69.3%
44.4	14.4	514	68	1889	8.6	2.31	0.6	0.17	17/1.5	69.4%
43.4	13.8	508	72	1879	8.5	2.08	0.6	0.16	17/1.5	69.7%
49.7	14.8	479	70.6	1768	9.6	2.25	0.7	0.14	17/1.5	70.8%
37.5	10	514	84	2358	8	1.84	0.5	0.12	17/1.5	72.5%
45	10.5	510	150	2053	6	0.62	0.6	0.12	17/1.5	74.5%
47.5	10.3	511	63	2577	9	2.88	0.7	0.12	17/1.5	75.5%
62:1.5 working/spec-off ratio—scaling not observed										
43.4	13.8	513	71	N/A	8.5	2.05	0.7	0.47	62/1.5	74.1%
43.4	13.8	515	88	N/A	8	1.72	0.7	0.45	62/1.5	74.1%
81:1.5 working/spec-off ratio—scaling observed										
44.2	14.1	518	85	N/A	8.4	2.12–2.48	0.7	0.14	81/1.5	74.4%
Additional tests—simulation of a second-stage EDR										
22.2	11.1	63.5	8.52	321	0.5	1.62	0.9	0.47	5/1.33	52.7%
22.8	7.8	92.6	13.3	324	2.5	3.5	0.9	0.22	17/1.5	68.5%

**Table 4:** Economic comparison of the tested EDR with commercial EDR in Stalowa Wola.

Parameter		EDR Tested in Łagisza Power Plant		Commercial EDR in Stalowa Wola Power Plant
		Average	Deep Demineralization	
Flow rate [L/h]	Diluate	43.2	45	74,900
	Concentrate	15.0	20.5	74,900 (circulated through the EDR)
				16,000 (removed from the system)
Conductivity [µS/cm]	Feed	509	511	450
	Diluate	74.3	17.9	85
	Concentrate	1754	1540	1350
Working time/spec-off time [min]		17/1.5	17/1.5	17/2.5
Recovery [%]		69.4	63.1	71.9
Pressure drop [bar]	Diluate	0.65	0.70	1.6
	Concentrate	0.15	0.12	
Energy consumption [kWh/m^3^]	DC for EDR	0.275	0.905	0.134
	Pumping	0.018	0.015	0.092
Investment costs [€/m^3^]		0.138	0.122	0.330
Overall desalination costs [€/m^3^]		0.170	0.223	0.355

**Table 5:** Sensitivity analysis of the calculated demineralization costs for the investigated case of novel EDR in Łagisza (“average” case, 74.3 μS/cm product conductivity, comparable to Stalowa Wola product conductivity of 85 μS/cm). The values of the base scenario for commercial EDR in Stalowa Wola as well as the ratio of the total costs estimated for EDR tested in Łagisza to the total costs of Stalowa Wola EDR are presented for comparison.

Analyzed Parameter		Łagisza [€/m^3^]			Stalowa Wola [€/m^3^]	Ratio of Łagisza to Stalowa Wola Total Costs		
		Base	Optimistic	Pessimistic		Base	Optimistic	Pessimistic
Electricity costs	OPEX	0.032	0.015	0.076	0.025	-	-	-
	CAPEX	0.138	0.138	0.138	0.330	-	-	-
	Total	0.170	0.152	0.214	0.355	0.48	0.43	0.60
Membrane costs	OPEX	0.032	0.032	0.032	0.025	-	-	-
	CAPEX	0.138	0.127	0.196	0.330	-	-	-
	Total	0.170	0.159	0.228	0.355	0.48	0.45	0.64
Equipment costs	OPEX	0.032	0.032	0.032	0.025	-	-	-
	CAPEX	0.138	0.113	0.162	0.330	-	-	-
	Total	0.170	0.145	0.194	0.355	0.48	0.41	0.55
Membrane lifetime	OPEX	0.032	0.032	0.032	0.025	-	-	-
	CAPEX	0.138	0.115	0.172	0.330	-	-	-
	Total	0.170	0.147	0.204	0.355	0.48	0.41	0.58
All factors simultaneously	OPEX	0.032	0.015	0.076	0.025	-	-	-
	CAPEX	0.138	0.085	0.275	0.330	-	-	-
	Total	0.170	0.100	0.351	0.355	0.48	0.28	0.99

**Table 6:** Sensitivity analysis of the calculated deep demineralization costs for the investigated case of novel EDR in Łagisza (“deep demineralization” case, 17.9 μS/cm product conductivity, significantly lower than the Stalowa Wola product conductivity of 85 μS/cm). The values of the base scenario for commercial EDR in Stalowa Wola as well as the ratio of the total costs estimated for EDR tested in Łagisza to the total costs of Stalowa Wola EDR are presented for comparison.

Analyzed Parameter		Łagisza [€/m^3^]			Stalowa Wola [€/m^3^]	Ratio of Łagisza to Stalowa Wola Total Costs		
		Base	Optimistic	Pessimistic		Base	Optimistic	Pessimistic
Electricity costs	OPEX	0.101	0.046	0.239	0.025	-	-	-
	CAPEX	0.138	0.138	0.138	0.330	-	-	-
	Total	0.239	0.184	0.377	0.355	0.67	0.52	1.06
Membrane costs	OPEX	0.101	0.101	0.101	0.025	-	-	-
	CAPEX	0.138	0.127	0.196	0.330	-	-	-
	Total	0.239	0.228	0.297	0.355	0.67	0.64	0.84
Equipment costs	OPEX	0.101	0.101	0.101	0.025	-	-	-
	CAPEX	0.138	0.113	0.162	0.330	-	-	-
	Total	0.239	0.214	0.263	0.355	0.67	0.60	0.74
Membrane lifetime	OPEX	0.101	0.101	0.101	0.025	-	-	-
	CAPEX	0.138	0.115	0.172	0.330	-	-	-
	Total	0.239	0.216	0.273	0.355	0.67	0.61	0.77
All factors simultaneously	OPEX	0.101	0.046	0.239	0.025	-	-	-
	CAPEX	0.138	0.085	0.275	0.330	-	-	-
	Total	0.239	0.131	0.514	0.355	0.67	0.37	1.45

## Data Availability

Data are available on request.

## Abbreviations

The following abbreviations are used in this manuscript:

COD	Chemical oxygen demand
DC	Direct current
ED	Electrodialysis
EDR	Electrodialysis reversal
RO	Reverse osmosis
TOC	Total organic carbon

## Appendix A

For the economic analysis, only data obtained when a 17 min working time was followed by a 1.5 min spec-off time was included (see Table 3). The goal was to calculate the process costs at the conditions that are the closest match to the Stalowa Wola industrial EDR plant (17 min working/2.5 spec-off), so cases with very short or very long working times were neglected, as were cases when two-stage electrodialysis was simulated. The procedure for calculating the costs was as follows (the input data are presented in Table 3):

1. Collect the following input data:
  - Number of membrane pairs, *n*, dimensionless; in our case, *n* = 8.
  - Operating current density, *i* [A/m^2^]; in our case, *i* = 8.57 A/m^2^ (average value of the 17/1.5 working/spec-off time ratio in Table 3) or *i* = 11.4 A/m^2^ (value that allowed for deep demineralization at a 17/1.5 working/spec-off time ratio).
  - Operating voltage per membrane pair, *U* [V]; *U* = 2.51 V and *U* = 7 V for the average case and deep demineralization case, respectively.
  - Electrode area, *A* [m^2^]. This value stems from the module geometry; in our case, *A* = 0.0836 m^2^.
  - Feed flow rate, *V_F_* [m^3^/h], which is the sum of the diluate flow rate *V_D_* and concentrate flow rate *V_C_*. In the average case, V_F_ = 58.15 L/h, V_D_ = 43.17 L/h, V_C_ = 14.98 L/h; in the deep demineralization case, V_F_ = 65.5 L/h, V_D_ = 45 L/h, V_C_ = 20.5 L/h.
  - Pressure drop along the diluate compartment; for the average case, Δ*P_D_* = 0.65 bar, and for the concentrate compartment, Δ*P_C_* = 0.153 bar; for the deep demineralization case, Δ*P_D_* = 0.7 bar, and for the concentrate compartment, Δ*P_C_* = 0.12 bar.
2. Calculate the energy required for electrodialysis as the DC current power per m^3^ of feed water treated, assuming a rectifier efficiency *η_AC_*_/_*_DC_* of 90% (the rectifier transforms AC current into DC current required by the EDR, but part of the energy is lost; 90% is a typical value in the industry). By definition, the power is voltage x current, so taking into account the number of membrane pairs and electrode area, the equation for the average case and the deep demineralization case becomes, respectively:
  (A1) E_DC_ = (n · U · A · i)/(η_AC/DC_ · V_F_) = (8 · 2.51 V · 0.0836 m^2^ · 8.57 A · m^−2^)/(0.9 · 58.15 dm^3^ · h^−1^) = 0.275 W · dm^−3^ · h = 0.275 kWh · m^−3^,
  (A2) E_DC_ = (n · U · A · i)/(η_AC/DC_ · V_F_) = (8 · 7 V · 0.0836 m^2^ · 11.4 A · m^−2^)/(0.9 · 65.5 dm^3^ · h^−1^) = 0.905 W · dm^−3^ · h = 0.905 kWh · m^−3^,
3. Calculate the energy required for pumping per 1 m^3^ of feed water. The power requirement for the pump is pressure drop x flow rate/pump efficiency, so if we include different pressure drops in the diluate and concentrate compartments and assume a pump efficiency *η_p_* of 80%, we get, for the average case and the deep demineralization case, respectively:
  (A3) E_AC_ = (V_D_ · ΔP_D_ + V_C_ · ΔP_C_)/(η_p_ · V_F_) = (43.17 dm^3^ · h^−1^ · 0.65 · 10^5^ Pa + 14.98 dm^3^ · h^−1^ · 0.153 · 10^5^ Pa)/(0.8 · 58.15 dm^3^ · h^−1^) = 0.018 kWh · m^−3^,
  (A4) E_AC_ = (V_D_ · ΔP_D_ + V_C_ · ΔP_C_)/(η_p_ · V_F_) = (40.5 dm^3^ · h^−1^ · 0.7 · 10^5^ Pa + 20.5 dm^3^ · h^−1^ · 0.12 · 10^5^ Pa)/(0.8 · 65.5 dm^3^ · h^−1^) = 0.015 kWh · m^−3^,
4. Next, calculate the operating costs by assuming that only the pumping costs and DC energy costs matter (chemicals costs are neglected) and assuming that the cost of electricity *C_el_* is 0.11 €/kWh. For the average case and the deep demineralization case, respectively, the result is:
  (A5) OPEX = C_el_ · (E_AC_ + E_DC_) = 0.11 · (0.018 + 0.275) = 0.032 € · m^−3^,
  (A6) OPEX = C_el_ · (E_AC_ + E_DC_) = 0.11 · (0.015 + 0.905) = 0.101 € · m^−3^,
5. Next, calculate the investment costs using the following assumptions:
  - Membrane cost of 38 €/m^2^ [31].
  - Only 75% (η_m_) of membrane area is the effective membrane area, and the remaining 25% is the sealing. Figure 4 presents the geometry of the spacer that can be used to explain the concept. Liquid can only flow along the white part in the middle, while the gray part is the sealing that prevents the liquid from spilling out. The membrane is in contact with the spacer and has the same area, but mass transfer can only occur when the membrane is in contact with the flowing liquid (i.e., only the area of the size of the white part in the middle would be effective). Additionally, because EDR requires both cation- and anion-exchange membranes, every cost relative to the membrane area should be multiplied by 2 × the number of membrane pairs.
  - EDR equipment cost of 321 € per m^2^ of the effective membrane area installed. This is a common method for estimating the cost of ED/EDR plants, as the capital costs are mainly determined by the required membrane area for a given feed and product concentration [34]. For example, Chehayeb et al. [40] assumed that the fixed cost of ED is directly proportional to the membrane area. Nayar et al. [30] gave a capital cost value of a high-salinity ED plant of 600 $/m^2^, out of which 222 $/m^2^ was the membrane and 378 $/m^2^ was everything else. Taking into account a currency exchange of 1$ = 0.85€, we assumed a value of 321 €/m^2^ for the EDR equipment. In practice, that value could be lowered, because the low salinity of the feed does not necessitate as durable materials as those assumed in [30]. For the average case and for the deep demineralization case, the results are, respectively:
  (A7) CAPEX = [2n · (C_m_ + C_eq_) · A/η_m_]/(t_life_ · V_F_) = [2 · 8 · (38 € · m^−2^ + 321 € · m^−2^) · 0.0836 m^2^/0.75]/(80000 h · 58.15 dm^3^ · h^−1^) = 0.138 € · m^−3^,
  (A8) CAPEX = [2n · (C_m_ + C_eq_) · A/η_m_]/(t_life_ · V_F_) = [2 · 8 · (38 € · m^−2^ + 321 € · m^−2^) · 0.0836 m^2^/0.75]/(80000 h · 65.5 dm^3^ · h^−1^) = 0.122 € · m^−3^,

The total costs are therefore 0.137 €/m^3^ + 0.032 €/m^3^ = 0.170 €/m^3^ for the average case and 0.122 €/m^3^ + 0.101 €/m^3^ = 0.223 €/m^3^ for the deep demineralization case.

For the Stalowa Wola case, the above described calculations can be repeated using the following input data, as reported by the industry: V_F_ = 90.9 m^3^/h, V_D_ = 74.9 m^3^/h, V_C_ = 74.9 m^3^/h (the system works in the feed-and-bleed mode on the concentrate side, 16 m^3^/h is removed from the system, the remaining 58.9 m^3^/h of concentrate is mixed with 16 m^3^/h of fresh feed and recycled to the EDR process), power consumption n · U · i · A = 11 kW, total area of installed membranes 2nA/η_m_ = 6689 m^2^, and pressure drop Δ*P_D_* = Δ*P_C_* = 1.6 bar.

(A9) E_DC_ = (n · U · A · i)/(η_AC/DC_ · V_F_) = (11 kW)/(0.9 · 90.9 m^3^ · h^−1^) = 0.134 kWh · m^−3^,

(A10) E_AC_ = (V_D_ · ΔP_D_ + V_C_ · ΔP_C_)/(η_p_ · V_F_) = (74.9 m^3^ · h^−1^ · 1.6 · 10^5^ Pa + 74.9 m^3^ · h^−1^ · 1.6 · 10^5^ Pa)/(0.8 · 90.9 m^3^ · h^−1^) = 0.092 kWh · m^−3^,

(A11) OPEX = C_el_ · (E_AC_ + E_DC_) = 0.11 € · kWh^−1^ · (0.092 kWh · m^−3^ + 0.134 kWh · m^−3^) = 0.025 € · m^−3^,

(A12) CAPEX = [(C_m_ + C_eq_) · (2nA/η_m_)]/(t_life_ · V_F_) = [(38 € · m^−2^ + 321 € · m^−2^) · 6689 m^2^]/(80000 h · 90.9 m^3^h^−1^) = 0.330 € · m^−3^,
